# Neonatal and maternal outcomes following transfer of vitrified embryos compared with slow-frozen and fresh embryos: a retrospective study in singletons

**DOI:** 10.1186/s12884-026-09493-4

**Published:** 2026-07-04

**Authors:** Yue Ying, Jianpeng Chen, Yun Huang, Xilin Shen, YiFeng Lin, Dan Zhang

**Affiliations:** 1 Department of Reproductive Endocrinology, Key Laboratory of Reproductive Genetics (Ministry of Education), Women’s Hospital, Zhejiang University School of Medicine, Hangzhou, 310006 Zhejiang China; 2 Key Laboratory of Women’s Reproductive Health of Zhejiang Province and Department of Reproductive Endocrinology, Women’s Hospital, Zhejiang University School of Medicine, Hangzhou, Zhejiang People’s Republic of China; 3 Zhejiang Provincial Clinical Research Center for Child Health, Zhejiang University School of Medicine, Hangzhou, Zhejiang People’s Republic of China

**Keywords:** Vitrification, Slow-freeze, Frozen embryo transfer, Fresh embryo transfer, Neonatal outcome, Maternal outcome

## Abstract

**Purpose:**

Embryo cryopreservation plays an essential role in assisted reproductive technology (ART). Vitrification has gradually replaced slow-freezing of embryos. In this study we investigated the effects of embryo vitrification on neonatal and maternal health.

**Methods:**

The retrospective study involved 19,752 in vitro fertilization (IVF) or intracytoplasmic sperm injection (ICSI) cycles performed at the Reproductive Medicine Center in Women’s Hospital, School of Medicine, Zhejiang University between October 2014 and September 2019. 7707 singletons got live birth, among which 427 were born after transfer of vitrified embryos, 3737 slow-frozen and 3543 fresh. Neonatal and maternal outcomes of singleton liveborns following transfer of vitrified were compared with slow-frozen and fresh embryos. Logistic regression analysis was conducted to adjust for possible confounders.

**Results:**

Transfer of vitrified embryos was comparable with slow-frozen embryos in low birth weight, macrosomia, small/large for gestational age (SGA/LGA) and birth defects. When compared with transfer of fresh embryos, transfer of vitrified embryos was associated with a higher risk of hypertensive disorders in pregnancy (HDP) aOR 7.12 (3.82–13.06), postpartum hemorrhage (PPH) aOR 6.81 (3.15–14.48) and cesarean section aOR 1.67 (1.34–2.10). No statistical differences were found for birth defects. There was a rising trend in birth weight, fetal macrosomia and LGA, as well as a declining trend in LGA after transfer of vitrified embryos compared to fresh embryos, though no statistical differences were found for risks.

**Conclusion:**

Transfer of vitrified embryos was comparable in birth weight and birth defects with slow-frozen embryos. When compared with fresh embryo transfer, transfer of vitrified embryos showed higher risks of HDP, PPH and cesarean section, and a rising trend in birth weight, which needs further follow-up.

**Trial Registration:**

IRB-20190052, 6th June 2019 retrospectively registered.

**Supplementary Information:**

The online version contains supplementary material available at https://doi.org/10.1186/s12884-026-09493-4.

## Introduction

Due to changes in embryo transfer policies and freeze-all strategies for various circumstances, cryopreservation has become an essential part of assisted reproductive technology (ART) [1]. In the field of ART, frozen embryo transfer (FET) is more advantageous than fresh embryo transfers in certain clinical situations and patient populations. For patients at risk of ovarian hyperstimulation syndrome or premature progesterone rise, when extra embryos are still available after fresh transfer, FET is frequently used [2–9]. Freeze-all strategies were reported to avoid induced hyperstimulation and increase live birth rates for women with high ovarian response or polycystic ovarian syndrome (PCOS), as well as reduce miscarriage rates for PCOS patients [10, 11]. The USA has witnessed a steady rise in the use of frozen embryos in ART cycles, as in 2020 81.6% of ART cycles utilized FET, up from 24.2% in 2010 [12, 13]. Trends like this are not just limited to the USA, but also across the globe. According to European researches, 35.5% of in vitro fertilization (IVF) or intracytoplasmic sperm injection (ICSI) cycles were finished with FETs in 2018 [14], up from 24.0% in 2008 [15].

As quantity of FETs grew, numerous studies have been carried out to compare FETs to fresh embryo transfers in terms of effectiveness as well as maternal and neonatal outcomes. FET has been linked to changed health outcomes [16–18]. Occurrences of all hypertensive disorders in pregnancy (HDP) increased in FET [19–21]. When Compareing with children born after fresh embryo transfer, children born after FET are at higher risks of macrosomia and large for gestational age (LGA), while faced lower risks of preterm birth and small for gestational age (SGA) [16, 19, 20, 22–25], which may elevate the chance of cesarean section, shoulder dystocia and postpartum hemorrhage [26]. Prior to being used on a broad scale, embryo vitrification should be thoroughly researched for safety.

In order to allow for cellular dehydration while minimize intracellular ice formation, a slow-freezing approach that uses propylene glycol and sucrose as a cryoprotectant has been utilized extensively for over 20 years. [27, 28]. In the last decade, human embryos are more frequently vitrified than slow-frozen previously. Vitrification is a cryopreservation technique that transforms the embryo into a glass-like condition without ice formation [29, 30]. In comparison with traditional slow-freezing technique, it is ultra-rapid and reduces cell damage [31]. In consequence, superior embryo cryo-survival rate and improved clinical results, including clinical pregnancy rate and live birth rate, were reported after embryo vitrification than slow-freezing [31–33]. Although it has been proposed that freeze–thaw methods may impair offspring health, research variability has made it difficult to draw conclusive conclusions on the impact of embryo vitrification. Majority of researches based on slow-freezing methods, while others describe results combining slow-freezing and vitrification methods [34, 35]. Rare are large studies that analyze perinatal outcomes following embryo vitrification [36, 37]. Few studies compared neonatal and maternal outcomes after vitrified, slow-frozen and fresh embryo transfer at the same time, which can provide more consolidated and valuable information.

In this single-center retrospective study, we analyzed neonatal and maternal outcomes including birth defects for pregnancies with singleton liveborns after transfer of vitrified embryos compared with slow-frozen and fresh embryos to investigate the effects of embryo vitrification on neonatal and maternal health.

## Methods

### Study design and participants

This retrospective study was approved by the ethnic committee in Women’s Hospital, School of Medicine, Zhejiang University (reference: IRB-20190052) and all data were collected from the electronic medical record system in the department of Reproductive Medicine Center. Couples who participated in IVF/ICSI embryo transfer cycles with autologous oocytes and sperms from October 2014 to September 2019 were analyzed. The exclusion criteria were as follows: 1) maternal age was over 45 years; 2) embryos experienced preimplantation genetic testing; 3) data were incomplete or inaccurate in the database. Singleton liveborns following transfer of vitrified and slow-frozen embryos were compared. Comparisons were also made between singleton liveborns following transfer of vitrified and fresh embryos. Live birth was defined as live infant born at 28 weeks or more of gestation.

### Controlled ovarian stimulation and embryo culture

The ovarian stimulation protocol, embryo culture protocol and embryo selection criteria have been summarized elsewhere [38, 39]. In short, patients were treated with follicle stimulating hormone (FSH) after gonadotropin releasing hormone (GnRH-a). Follicular development was monitored using serial vaginal ultrasound and serum E2 levels. Human chorionic gonadotropin (hCG) was administered when two or more follicles reached 18 mm in mean diameter. Oocytes were transvaginally retrieved under ultrasound guidance. The number of oocytes retrieved at one time was usually between 8 and 15. Oocytes, zygotes and embryos were cultured in G-series reagents (Vitrolife Sweden AB, Västra Frölunda, Sweden) at 37 °C in a humidified atmosphere containing 6% CO2. Embryos were cultured in vitro for 3–5 days.

### Freezing, thawing and transfer of embryos

For slow freezing, cleavage-stage embryos were frozen using a Planer freezer (Planer Ltd, Sunbury, Middlesex, UK) and the Embryo Freeze Media Kit (Irvine Scientific, Santa Ana, CA, USA). The Embryo Thaw Media Kit (Irvine Scientific, Santa Ana, CA, USA) was used for embryo warming. The slow freezing programme was conducted according to the manufacturers’instructions, and the specific steps have been described elsewhere [38].

For vitrification, cleavage-stage embryos were frozen using Cryotop strip for open vitrification procedure in combination with ethylene glycol-dimethylsulfoxide-sucrose (Kitazato Supply Co) as the cryoprotectant.

The choice of cryopreservation method (vitrified or slow-frozen) is random.

Embryos were transferred during controlled ovarian hyperstimulation (COH) or oestrogen–progesterone hormonally supplemented cycles. Before transfer, the warmed embryos were cultured in G-1 (Vitrolife Sweden AB, Västra Frölunda, Sweden) medium for at least 2 h. One to three embryos were transferred for all patients. After transfer, luteal support is applied.

### Outcomes

The main outcomes were birth weight, low birth weight (birth weight < 2500 g), macrosomia (birth weight ≥ 4000 g), SGA and LGA. SGA was defined as weighing less than the 10th percentile for the gestational age and sex. The definition of LGA was the birth weight greater than 90th percentile for the gestational age and sex. This study uesd the 2014 reference of Chinese infants from 28 to 44 gestation weeks as the reference [40].

Secondary outcomes were gestational age (week), premature birth (gestational age < 37 weeks), overdue delivery (gestational age ≥ 42 weeks), any birth defect (International Statistical Classification of Diseases and Related Health Problems (ICD)−10 codes beginning with Q), GDM (gestational diabetes mellitus) (ICD-10 code O24), HDP (pregnancy-induced hypertension) (ICD-10 code O13-O15), PROM(premature rupture of membrane) (ICD-10 code O42), PPH (postpartum hemorrhage) (ICD-10 code O72) and cesarean section (ICD-10 code O82). Birth defect was identified by birth hospital or pediatric care facility, and was monitored by a skilled nurse until the child was three years old.

### Statistical analyses

Statistical analyses were performed with R-4.2.0. All continuous variables were presented as the mean ± SD and compared by means of analysis of variance and the Bonferroni test was used for pairwise comparison. Categorical variables were presented as frequencies and percentages and compared by means of chi-square tests. Multivariable logistic (for categorical variables) and linear (for continuous variables) regression analyses were conducted and adjustment was made for female age, male age, female BMI, cause of infertility and ART method, number of embryos transfered, latest stage of embryos transfered and quality of embryo transferred. For each outcome, crude and adjusted odds ratios (aORs) with 95% confidence intervals (CIs) and *P* value were computed. Missing data was not substituted.

## Results

**Fig. 1:**
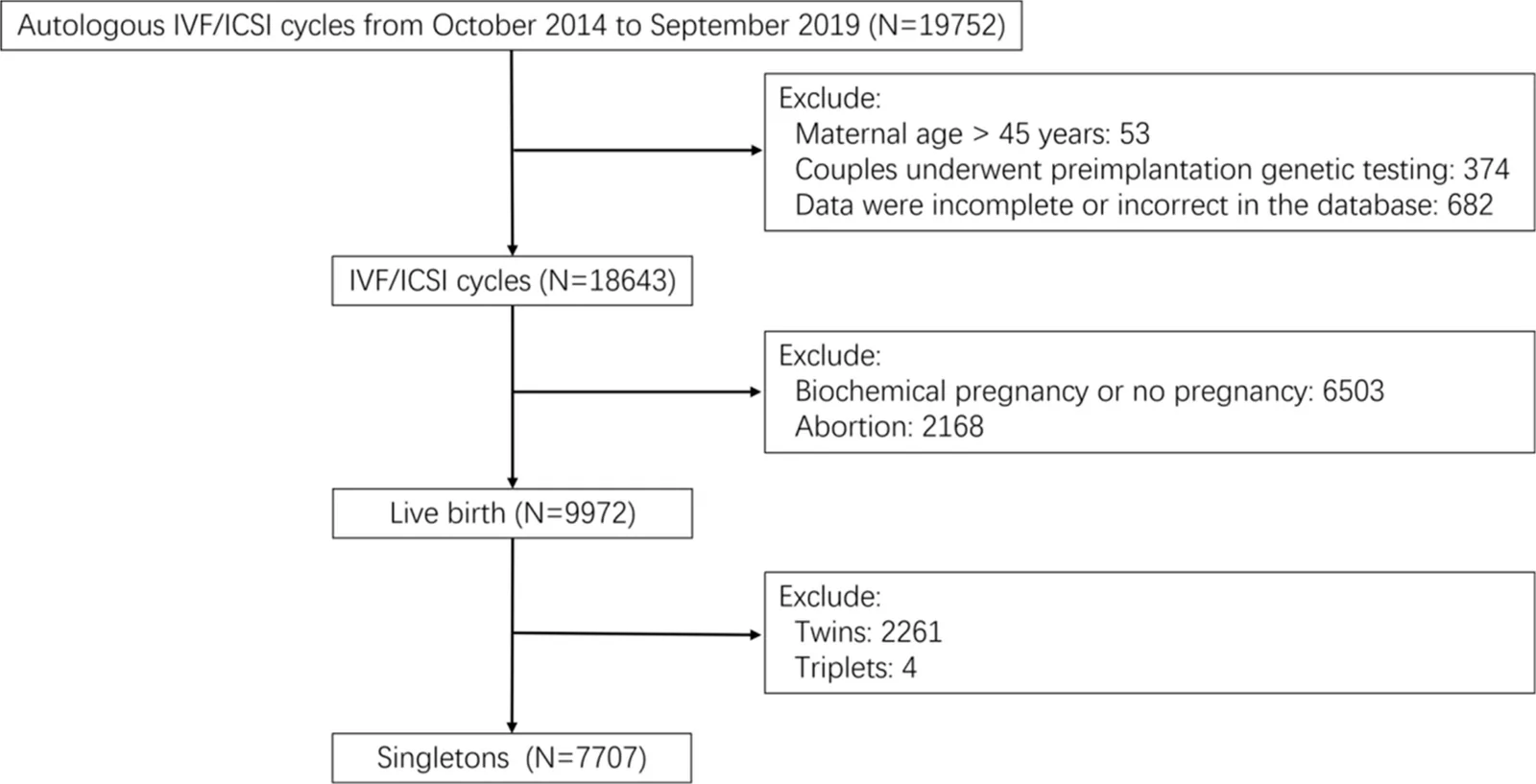
Flowchart of the study population. Abbreviations: IVF, in vitro fertilization; ICSI, intracytoplasmic sperm injection

The study involved 19,752 IVF/ICSI cycles. Clinical pregnancy rate were showed in Table S1. In total, 7707 singleton live births were involved (Fig. 1), among which 427 singletons were born after the transfer of vitrified embryos, 3737 after slow-frozen embryo transfer and 3543 after fresh embryo transfer. Table 1 displayed the baseline characteristics of the enrolled couples who gave birth to singletons following vitrified embryo transfer, comparing with slow-frozen and fresh embryo transfer from October 2014 to September 2019.

**Table 1:** Baseline characteristics of the included couples giving birth to singletons conceived after vitrified embryo transfer compared with slow-frozen and fresh embryo transfer, 2014–2019

Variable^a^	Vitrified	Slow-frozen	Fresh	Test between groups, *P*-value^b^	
				Vitrified vs. slow-frozen	Vitrified vs. fresh
No. of cycles	427	3737	3543		
Female age (y)	29.68±3.86	30.36±4.05	30.85±3.98	**0.003**	**< 0.001**
Male age (y)	31.51±4.65	32.35±5.04	32.68±4.93	**0.003**	**0.016**
Female BMI (kg/m2)	21.28±2.46	21.84±2.69	21.95±2.68	**< 0.001**	0.290
Male BMI (kg/m2)	24.2±3.42	23.89±3.49	23.92±3.54	0.262	1.000
Type of infertility				0.662	0.092
Primary	202 (47.31)	1810 (48.42)	1625 (45.87)		
Secondary	225 (52.69)	1928 (51.58)	1918 (54.13)		
Duration of infertility (y)	3.18±2.42	3.5±2.72	3.47±2.70	0.063	1.000
Cause of infertility					
Ovulatory dysfunction	53 (12.41)	365 (9.79)	190 (5.37)	0.088	**< 0.001**
Diminished ovarian reserve	18 (4.22)	184 (4.93)	211 (5.96)	0.513	0.085
Endometriosis	15 (3.51)	211 (5.66)	268 (7.57)	0.064	**< 0.001**
Tubal factor	246 (57.61)	2164 (58.03)	2010 (56.76)	0.868	0.549
Uterine factor	14 (3.28)	87 (2.33)	104 (2.94)	0.229	0.200
Male factor	53 (12.41)	483 (12.95)	526 (14.85)	0.752	**0.044**
Unexplained	28 (6.56)	235 (6.3)	232 (6.55)	0.837	0.905
ART method				**0.002**	**0.008**
IVF	294 (71.88)	2364 (64.15)	2261 (64.67)		
ICSI	115 (28.12)	1321 (35.85)	1235 (35.33)		
Number of embryos transfered				**< 0.001**	**< 0.001**
1	256 (59.95)	793 (21.22)	734 (20.72)		
2	170 (39.81)	2878 (77.01)	2756 (77.79)		
3	1 (0.23)	66 (1.77)	53 (1.50)		
Latest stage of embryos transferred				**< 0.001**	**< 0.001**
Cleavage	343 (80.90)	2199 (59.24)	3541 (99.97)		
Morula or early blastocyst	81 (19.10)	1513 (40.76)	1 (0.03)		
Quality of embryo transferred				**< 0.001**	**< 0.001**
all of good quality	249 (58.73)	2220 (59.81)	2677 (75.58)		
mix of good and poor quality	152 (35.85)	1026 (27.64)	276 (7.79)		
all of poor quality	23 (5.42)	466 (12.55)	589 (16.63)		
Endometrial preparation				0.223	
Natural cycle	109 (26.01)	991 (26.91)		0.696	
Hormone replacement cycle	261 (62.29)	2357 (64)		0.491	
Stimulated cycle	49 (11.69)	335 (9.1)		0.084	

*Abbreviations*: *BMI* body mass index, *y* year, *ART* assisted reproductive technology, *IVF*, in vitro fertilization, *ICSI* intracytoplasmic sperm injection

^a^Values are presented as mean ± SD or frequency (percentage)

^b^*P* value is based on Bonferroni for continuous variables and χ2 test for categorical variables. Results in bold indicate statistical significance (*P* < 0.05)

Neonatal and maternal outcomes in pregnancies with singleton liveborns following vitrified embryo transfer comparing to slow-frozen and fresh embryo transfer along with statistical analysis are summarised in Tables 2, 3 and 4. The comparison to slow-frozen embryo transfer and fresh embryo transfer were in Table S2 [24].

**Table 2:** Neonatal and maternal outcome in pregnancies with singleton liveborns after vitrified embryo transfer compared with slow-frozen and fresh embryo transfer, 2014–2019

Variable^a^	Vitrified	Slow-frozen	Fresh	Test between groups, *P*-value^b^	
				Vitrified vs. slow-frozen	Vitrified vs. fresh
No. of liveborns	427	3737	3543		
Gestational age (wk)	38.39 ± 1.81	38.44 ± 1.75	38.52 ± 1.72	> 0.999	0.207
Premature birth	45 (10.54)	352 (9.42)	300 (8.47)	0.456	0.199
Overdue delivery	0 (0)	7 (0.19)	10 (0.28)	> 0.999	0.594
Gender				0.087	0.229
Boys	242 (56.67)	1952 (52.23)	1863 (52.64)		
Girls	185 (43.33)	1780 (47.63)	1676 (47.36)		
Birth weight (g)	3290.53 ± 521.51	3297.22 ± 520.67	3271.57 ± 523.30	> 0.999	0.110
Birth weight (g, boys)	3346.47 ± 502.86	3350.9 ± 519.76	3315.28 ± 525.34	> 0.999	0.106
Birth weight (g, girls)	3217.65 ± 537.53	3238.3 ± 515.56	3222.7 ± 516.93	> 0.999	> 0.999
Low birth weight infant	27 (6.34)	207 (5.56)	214 (6.08)	0.512	0.584
Low birth weight infant (boys)	11 (4.56)	95 (4.88)	105 (5.66)	0.830	0.496
Low birth weight infant (girls)	16 (8.65)	112 (6.32)	109 (6.55)	0.223	0.476
Fetal macrosomia	31 (7.28)	289 (7.77)	243 (6.91)	0.719	0.371
Fetal macrosomia (boys)	24 (9.96)	187 (9.6)	149 (8.04)	0.861	0.199
Fetal macrosomia (girls)	7 (3.78)	102 (5.76)	94 (5.65)	0.266	0.537
SGA	21 (4.93)	213 (5.70)	260 (7.39)	0.498	**0.007**
LGA	66 (15.49)	590 (15.79)	509 (14.47)	0.840	0.251
Birth defects, any	5 (1.17)	70 (1.87)	62 (1.75)	0.301	0.574
GDM	13 (3.04)	116 (3.10)	122 (3.44)	0.946	0.694
HDP	21 (4.92)	114 (3.05)	30 (0.85)	**0.039**	**< 0.001**
PROM	31 (7.26)	138 (3.69)	66 (1.86)	**< 0.001**	**< 0.001**
PPH	14 (3.28)	47 (1.26)	19 (0.54)	**0.001**	**< 0.001**
Cesarean section	275 (64.4)	2471 (66.12)	1941 (54.85)	0.468	** < 0.001**

*Abbreviations*: *SGA/LGA* small/large for gestational age, *GDM* gestational diabetes mellitus, *HDP* hypertensive disorders in pregnancy, *PROM* premature rupture of membrane, *PPH* postpartum hemorrhage

^a^Values are presented as mean ± SD or frequency (percentage)

^b^*P* value is based on Bonferroni for continuous variables and χ2 test for categorical variables. Results in bold indicate statistical significance (*P* < 0.05)

**Table 3:** Crude and adjusted odds ratios of neonatal and maternal outcome in pregnancies with singleton liveborns after vitrified embryo transfer compared with slow-frozen and fresh embryo transfer in logistic regression

	Vitrified vs. slow-frozen				Vitrified vs. fresh			
	Crude OR (95% CI)	*P* value	Adjusted OR (95% CI)	*P* value	Crude OR (95% CI)	*P* value	Adjusted OR (95% CI)	*P* value
Premature birth	1.13 (0.81–1.56)	0.456	1.14 (0.80–1.60)	0.444	1.27 (0.90–1.76)	0.152	1.29 (0.90–1.81)	0.158
Overdue delivery	-	-	-	-	-	-	-	-
Gender (boys)	1.19 (0.98–1.46)	0.087	1.19 (0.97–1.48)	0.103	1.18 (0.96–1.44)	0.115	1.15 (0.93–1.44)	0.196
Low birth weight infant	1.15 (0.74–1.71)	0.513	1.22 (0.78–1.85)	0.363	1.05 (0.68–1.55)	0.834	1.07 (0.68–1.62)	0.776
Low birth weight infant (boys)	0.93 (0.47–1.69)	0.830	0.95 (0.46–1.77)	0.876	0.80 (0.40–1.44)	0.484	0.78 (0.38–1.47)	0.47
Low birth weight infant (girls)	1.40 (0.78–2.36)	0.225	1.53 (0.84–2.63)	0.145	1.35 (0.75–2.27)	0.283	1.42 (0.76–2.50)	0.244
Fetal macrosomia	0.93 (0.62–1.35)	0.719	1.05 (0.69–1.55)	0.807	1.06 (0.70–1.54)	0.776	1.18 (0.77–1.77)	0.424
Fetal macrosomia (boys)	1.04 (0.65–1.60)	0.861	1.23 (0.74–1.95)	0.402	1.27 (0.79–1.96)	0.309	1.44 (0.86–2.32)	0.15
Fetal macrosomia (girls)	0.64 (0.27–1.31)	0.269	0.69 (0.28–1.43)	0.356	0.66 (0.27–1.34)	0.293	0.72 (0.29–1.52)	0.427
SGA	0.85 (0.52–1.32)	0.499	0.89 (0.53–1.40)	0.621	0.65 (0.40–1.00)	0.064	0.68 (0.41–1.09)	0.127
LGA	0.97 (0.73–1.27)	0.840	1.05 (0.78–1.40)	0.739	1.08 (0.81–1.42)	0.573	1.17 (0.86–1.58)	0.307
Birth defects, any	0.62 (0.22–1.40)	0.306	0.64 (0.22–1.48)	0.347	0.67 (0.23–1.51)	0.383	0.71 (0.24–1.67)	0.475
GDM	0.98 (0.52–1.69)	0.946	0.92 (0.46–1.68)	0.805	0.88 (0.47–1.51)	0.668	0.69 (0.34–1.28)	0.271
HDP	1.64 (0.99–2.59)	**0.041**	**1.98 (1.15–3.27)**	**0.010**	**6.06 (3.39–10.62)**	**< 0.001**	**7.24 (3.85–13.4)**	**< 0.001**
PROM	**2.04 (1.34–3.01)**	**0.001**	**1.85 (1.17–2.84)**	**0.006**	**4.12 (2.63–6.34)**	** < 0.001**	**3.22 (1.95–5.22)**	**< 0.001**
PPH	**2.66 (1.40–4.75)**	**0.002**	**2.45 (1.23–4.63)**	**0.008**	**6.29 (3.07–12.57)**	**< 0.001**	**6.24 (2.85–13.42)**	**< 0.001**
Cesarean section	0.93 (0.75–1.14)	0.468	1.02 (0.82–1.28)	0.869	**1.49 (1.21–1.84)**	**< 0.001**	**1.75 (1.40–2.20)**	** < 0.001**

^a^Odds ratio, 95% confidence interval (CI) and P value were calculated from multivariable logistic regression models. Results in bold indicate statistical significance

^b^Adjusted models are controlled for female age, male age, female BMI, cause of infertility and ART method, number of embryos transfered and latest stage of embryos transfered

**Table 4:** Crude and adjusted odds ratios of neonatal and maternal outcome in pregnancies with singleton liveborns after vitrified embryo transfer compared with slow-frozen and fresh embryo transfer in linear regression

	Vitrified vs. slow-frozen				Vitrified vs. fresh			
	Crude β (95% CI)	*P* value	Adjusted β (95% CI)	*P* value	Crude β (95% CI)	*P* value	Adjusted β (95% CI)	*P* value
Gestational age (wk)	−0.02 (−0.19 to 0.16)	0.551	−0.01 (−0.2 to 0.17)	0.634	−0.04 (−0.21 to 0.14)	0.153	−0.03 (−0.22 to 0.15)	0.226
Birth weight (g)	−0.01 (−52.34 to 52.33)	0.802	0 (−55.01 to 55.01)	0.955	0.02 (−52.47 to 52.5)	0.479	0.03 (−56.01 to 56.07)	0.259
Birth weight (g, boys)	0 (−69.8 to 69.79)	0.901	0.02 (−73.45 to 73.49)	0.583	0.03 (−69.96 to 70.02)	0.382	0.05 (−74.37 to 74.47)	0.175
Birth weight (g, girls)	−0.02 (−78.39 to 78.35)	0.605	−0.02 (−82.22 to 82.17)	0.548	0 (−78.61 to 78.6)	0.900	0 (−84.31 to 84.32)	0.922

^a^Beta coefficients, 95% confidence interval (CI) and P value were calculated from multivariable linear regression models. Results in bold indicate statistical significance

^b^Adjusted models are controlled for female age, male age, female BMI, cause of infertility and ART method, number of embryos transfered and latest stage of embryos transfered

### Transfer of vitrified embryos versus slow-frozen embryos

For neonatal outcomes, there were no significant differences found for gestational age, premature birth, overdue delivery, gender, birth weight, low birth weight infant (boys or girls), fetal macrosomia (boys or girls), SGA, LGA and any birth defects.

For maternal outcomes, transfer of vitrified embryos was linked to an increased risk of HDP aOR (95%) 1.98 (1.15–3.25) P 0.017, PROM aOR (95%) 1.90 (1.21–2.91) P 0.004 and PPH aOR (95%) 2.51 (1.26–4.75) P 0.006, comparing with transfer of slow-frozen embryos. There were no significant differences found for GDM and cesarean section.

### Transfer of vitrified embryos versus fresh embryos

For neonatal outcomes, there were no statistical differences in risks of gestational age, premature birth, overdue delivery, gender, birth weight, low birth weight infant (boys or girls), fetal macrosomia (boys or girls), SGA, LGA and any birth defects after transfer of vitrified embryos comparing with transfer of fresh embryos. But there was a rising trend in birth weight, fetal macrosomia and LGA, as well as a declining trend in LGA after transfer of vitrified embryos compared to fresh embryos.

For maternal outcomes, transfer of vitrified embryos was linked to an increased risk of HDP aOR (95%) 7.12 (3.82–13.06) *P* < 0.001, PROM aOR (95%) 3.66 (2.24–5.86) *P* < 0.001, PPH aOR (95%) 6.81 (3.15–14.48) *P* < 0.001 and cesarean section aOR (95%) 1.67 (1.34–2.10) *P* < 0.001 comparing with transfer of fresh embryos. There were no significant differences found for GDM.

## Discussion

In this single-center retrospective study, we reported comparable outcomes for singletons born following transfer of vitrified embryos, in terms of premature birth, overdue delivery, birth weight, birth defects and cesarean section, with peers born after slow-frozen embryo transfer. Comparing with transfer of fresh embryos, vitrified embryos are linked to an increased risks of HDP, PPH and cesarean section and a rising trend in birth weight.

Between transfer of vitrified and slow-frozen embryos, there were no significant differences found for premature birth, overdue delivery, low birth weight infant (boys or girls), fetal macrosomia (boys or girls), SGA, LGA and any birth defects. Currently, a few retrospective studies have tackled this problem by contrasting two freezing techniques with varying results. Numerous studies imply that the freezing method may not have an impact on perinatal outcomes. These studies did not find any differences in the risk of PTB, birthweight, SGA, LGA, or birth defects between vitrified and slow-frozen embryos in singleton pregnancies [33, 41, 42]. We saw a slight rise in transfer of vitrified embryos regarding HDP, PROM and PPH comparing with transfer of slow-frozen embryos. There were no significant differences found for GDM and cesarean section. In Ginström Ernstad E et al. and Wikland et al.’s study, compared with transfer of slow-frozen cleavage stage embryos, vitrified blastocysts showed no significant differences in HDP, placenta previa and placental abruption [42, 43]. But Wikland et al. found an increased risk of major post-partum haemorrhage associating with the vitrified blastocyst group [42].

Consistent with several previous studies demonstrating larger newborns after FET [16, 17, 22, 23, 37, 42–45], our findings revealed a rising trend in birth weight, fetal macrosomia and LGA, as well as a declining trend in LGA after transfer of vitrified embryos compared to fresh embryos, though no statistical differences in risks were found. Slight weight differences at birth are unlikely to have any negative effects on the progeny. Additionally, there is proof that the ponderal index of FET babies is comparable to that of fresh ET and NC newborns, indicating that FET children are just big in size rather than being "fat" [46]. Furthermore, there has been a concern raised over if the gender of the children affects the influences of the freeze–thaw process. Based on our research, we didn’t find significant difference in birthweight between boys and girls, in accordance with a retrospective Nordic register-based cohort research that found no sex-dependent variations in the aOR for LGA in the whole study population or in the term-born subgroup [47]. However, in a recent single-center study of 1295 singletons, it was found that term boys born following frozen blastocyst transfers had a significantly higher risk of LGA comparing to girls [48]. Moreover, FET was associated with a higher birthweight of term boys comparing to girls in a nationwide cohort of 180,184 singletons [45]. But Keane et al. found the effect of FET on birthweight was only significant for female newborns in their single-center research with stratification in Australia, with no apparent effect of FET on birthweight for male newborns [49]. Also in a single-center study, Kaartinen et al. found FET affected more significantly on female infants than on male infants for embryos transferred on 2–3 days and trends of higher birthweight in male infants following transfers on 5–6 days but no difference in female infants for the later transfer group [50].

Neonates following vitrified-warmed embryo transfer were comparable to those following fresh embryo transfer concerning preterm delivery rate, in accordance with previous observational studies [37, 51]. The major congenital malformation rates in singleton liveborns was similar between the vitrified group and the fresh group, which is consistent with results from most previous observational studies and meta-analyses [16, 24, 37, 44, 52–54]. Nevertheless, there are studies suggesting that the incidence of congenital malformations rise after FET, for digestive and facial malformations [55].

We observed an increased rate of hypertensive disorders in pregnancy (HDP), post-partum haemorrhage (PPH) and cesarean section in the vitrified group when comparing with the fresh groups. Lots of earlier study also found increased risk of HDP [36, 43, 51, 56–59] and PPH [16, 17, 42, 60]. Larger neonates and increased rates of cesarean section may make a contribution to the greater risk of PPH after transfer of vitrified embryos versus fresh embryos [26, 61]. Therefore, although FET can reduce the incidence of OHSS in patients with high ovarian response and achieve a better pregnancy rate, it may cause potential risks to the fetus and mother. Hence it is necessary to make embryo freezing decisions based on the advantages and risks of FET in clinical practice.

The increased risks of HDP in FET may also be owing to endometrial preparation. As previous study reported endometrial preparation protocols, particularly hormone replacement cycles, showed significantly higher rates of HDP and cesarean section [62]. The reason may be related to abnormal function of the vascular endothelium. Exogenous hormones (such as estrogen and progesterone) may interfere with the vascular remodeling process during pregnancy, resulting in insufficient placental perfusion. Placental ischemia can trigger oxidative stress and inflammatory responses, thereby increasing the risk of gestational hypertension disorders. However, higher rates of low birthweight was observed in hormone replacement cycles [62], which doesn’t match our findings. This suggests that the rising birth weight of FET might be due to factors except for endometrial preparation. In our baseline characteristics, we found no statistically significant difference of endometrial preparation plan of FET between the vitrified and slow-frozen groups. Further researches including prospective clinical trial and animal experimentation need to be conducted to clarify the impact.

In this research, transfer of vitrified, slow-frozen and fresh embryos were all performed at the same facility and compared together, which had been done in few studies. Because several other confounding factors were held constant and with a relatively large sample capacity, the comparisons here were meaningful. One limitation of the research was that embryo vitrification gradually increased in these years. So the difference of time span may cause some bias. And there were some clinical outcomes with actual low frequency, in which bias may be more likely to occur. What’s more, the present research is a single-center retrospective study. In the future, we tend to conduct multi-center prospective cohort study to provide more solid result.

There are probably multiple factors contributing to the different perinatal and maternal outcomes following transfer of vitrified embryos. There is no clear explanation yet for the observed discrepancies. Although epigenetic changes brought on by vitrification have been reported [63], it has also been discovered that the supra-physiologic hormonal environment present during ovarian stimulation in fresh embryo transfer cycles has a negative impact on children's health [64]. In animal embryos, vitrification can have a considerable impact on the expression and methylation of some imprinted genes, but there have been almost no influence in the few studies in humans [65]. The physical processes of freezing and thawing embryos may filter out weaker embryos and enable only high-quality ones to survive, resulting in better fetal development, which is another hypothesis put forth for better outcomes in pregnancies following vitrified transfer [66]. To determine how vitrification affects epigenetic markers and embryonic developmental competency, further researches are required.

## Conclusion

Transfer of vitrified embryos was comparable in premature birth, overdue delivery, birth weight, birth defects and cesarean section with transfer of slow-frozen embryos. When comparing with fresh embryo transfer, transfer of vitrified embryos showed an increased risks of HDP, PPH and cesarean section and a rising trend in birth weight. The above results provided some factors to consider for freeze-all strategy and offered some evidence for clinical decision made for embryo freezing. The safety of embryo vitrification still needs to be further assessed by long-term offspring follow-up.

## Supplementary Information

Supplementary Material 1.

Supplementary Material 2.

## Data Availability

The data and materials of the present study are available from the corresponding authors on reasonable request. reasonable request.
