# Supplementary material for: Neonatal and maternal outcomes following transfer of vitrified embryos compared with slow-frozen and fresh embryos: a retrospective study in singletons
Source: BMC Pregnancy Childbirth. 2026 Jul 4;26:1058. doi: 10.1186/s12884-026-09493-4 (PMC13613727; doi:10.1186/s12884-026-09493-4)
Supplement: Supplementary file 1 [file 12884_2026_9493_MOESM1_ESM.pdf]

Table S1. Clinical pregnancy rate after vitrified, slow-frozen and fresh embryo transfer, 2014-2

Variable	Vitrified	Slow-frozen	Fresh
No. of cycles	986	8789	8868
No. of clinical pregnancy	607	5857	5676
clinical pregnancy rate (%)	61.56	66.64	64.01

2019.
