# Supplementary material for: Neonatal and maternal outcomes following transfer of vitrified embryos compared with slow-frozen and fresh embryos: a retrospective study in singletons
Source: BMC Pregnancy Childbirth. 2026 Jul 4;26:1058. doi: 10.1186/s12884-026-09493-4 (PMC13613727; doi:10.1186/s12884-026-09493-4)
Supplement: Supplementary file 2 [file 12884_2026_9493_MOESM2_ESM.pdf]

Table S2. Neonatal and maternal outcome in pregnancies with singleton liveborns after vitrified embryo transfer compared with slow-frozen and fresh embryo transfer, and slow-frozen embryo transfer compared with fresh embryo transfer, 2014-2019.

Variable[a]	Vitrified	Slow-frozen	Fresh	Test between groups, *P*-value[b]		
				Vitrified vs. slow-frozen	Vitrified vs. fresh	Slow-frozen vs. fresh
No. of liveborns	427	3737	3543			
Gestational age (wk)	38.39±1.81	38.44±1.75	38.52±1.72	> 0.999	0.207	0.203
Premature birth	45 (10.54)	352 (9.42)	300 (8.47)	0.456	0.199	0.157
Overdue delivery	0 (0)	7 (0.19)	10 (0.28)	> 0.999	0.594	0.401
Gender				0.087	0.229	0.772
Boys	242 (56.67)	1952 (52.23)	1863 (52.64)			
Girls	185 (43.33)	1780 (47.63)	1676 (47.36)			
Birth weight (g)	3290.53±521.51	3297.22±520.67	3271.57±523.30	> 0.999	0.110	0.075
Birth weight (g, boys)	3346.47±502.86	3350.9±519.76	3315.28±525.34	> 0.999	0.106	0.083
Birth weight (g, girls)	3217.65±537.53	3238.3±515.56	3222.7±516.93	> 0.999	> 0.999	0.946
Low birth weight infant	27 (6.34)	207 (5.56)	214 (6.08)	0.512	0.584	0.345
Low birth weight infant (boys)	11 (4.56)	95 (4.88)	105 (5.66)	0.830	0.496	0.277
Low birth weight infant (girls)	16 (8.65)	112 (6.32)	109 (6.55)	0.223	0.476	0.780
Fetal macrosomia	31 (7.28)	289 (7.77)	243 (6.91)	0.719	0.371	0.135
Fetal macrosomia (boys)	24 (9.96)	187 (9.6)	149 (8.04)	0.861	0.199	0.079
Fetal macrosomia (girls)	7 (3.78)	102 (5.76)	94 (5.65)	0.266	0.537	0.836
SGA	21 (4.93)	213 (5.70)	260 (7.39)	0.498	**0.007**	**0.004**
LGA	66 (15.49)	590 (15.79)	509 (14.47)	0.840	0.251	0.087
Birth defects, any	5 (1.17)	70 (1.87)	62 (1.75)	0.301	0.574	0.629
GDM	13 (3.04)	116 (3.10)	122 (3.44)	0.946	0.694	0.452
HDP	21 (4.92)	114 (3.05)	30 (0.85)	**0.039**	**< 0.001**	**< 0.001**
PROM	31 (7.26)	138 (3.69)	66 (1.86)	**< 0.001**	**< 0.001**	**< 0.001**
PPH	14 (3.28)	47 (1.26)	19 (0.54)	**0.001**	**< 0.001**	**0.001**
Cesarean section	275 (64.4)	2471 (66.12)	1941 (54.85)	0.468	**< 0.001**	**< 0.001**

Abbreviations: SGA/LGA, small/large for gestational age; GDM, gestational diabetes mellitus; HDP, hypertensive disorders in pregnancy; PROM, premature rupture of membrane; PPH, postpartum hemorrhage.

a Values are presented as mean ± SD or frequency (percentage).

b P value is based on Bonferroni for continuous variables and χ^2 test for categorical variables. Results in bold indicate statistical significance ($P < 0.05$).
